# Mitophagy: A potential therapeutic target for insulin resistance

**DOI:** 10.3389/fphys.2022.957968

**Published:** 2022-08-23

**Authors:** Peng Ning, Xiaobo Jiang, Jing Yang, Jiaxing Zhang, Fan Yang, Hongyi Cao

**Affiliations:** ^1^ Department of Endocrine and Metabolism, Geriatric Diseases Institute of Chengdu/Cancer Prevention and Treatment Institute of Chengdu, Chengdu Fifth People’s Hospital(The Second Clinical Medical College, Affiliated Fifth People’s Hospital of Chengdu University of Traditional Chinese Medicine), Chengdu, China; ^2^ Department of Cardiovascular Medicine, Geriatric Diseases Institute of Chengdu/Cancer Prevention and Treatment Institute of Chengdu, Chengdu Fifth People’s Hospital(The Second Clinical Medical College, Affiliated Fifth People’s Hospital of Chengdu University of Traditional Chinese Medicine), Chengdu, China

**Keywords:** mitophagy, autophagy, mitochondrial dysfunction, insulin resistance, therapeutic target

## Abstract

Glucose and lipid metabolism disorders caused by insulin resistance (IR) can lead to metabolic disorders such as diabetes, obesity, and the metabolic syndrome. Early and targeted intervention of IR is beneficial for the treatment of various metabolic disorders. Although significant progress has been made in the development of IR drug therapies, the state of the condition has not improved significantly. There is a critical need to identify novel therapeutic targets. Mitophagy is a type of selective autophagy quality control system that is activated to clear damaged and dysfunctional mitochondria. Mitophagy is highly regulated by various signaling pathways, such as the AMPK/mTOR pathway which is involved in the initiation of mitophagy, and the PINK1/Parkin, BNIP3/Nix, and FUNDC1 pathways, which are involved in mitophagosome formation. Mitophagy is involved in numerous human diseases such as neurological disorders, cardiovascular diseases, cancer, and aging. However, recently, there has been an increasing interest in the role of mitophagy in metabolic disorders. There is emerging evidence that normal mitophagy can improve IR. Unfortunately, few studies have investigated the relationship between mitophagy and IR. Therefore, we set out to review the role of mitophagy in IR and explore whether mitophagy may be a potential new target for IR therapy. We hope that this effort serves to stimulate further research in this area.

## Introduction

Insulin resistance (IR) is a pathological condition that develops in response to genetic and environmental factors. IR describes a state of decreased uptake and utilization of glucose and a reduced response and sensitivity to insulin in the body (Lee S. E. et al., 2022). Disorders of glucose and lipid metabolism are critically important as they can lead to diabetes, coronary heart disease, obesity, metabolic syndrome, and other metabolic disorders; the incidences of these disorders have been rapidly increasing. IR is an important public health concern that imposes a significant health care burden on society (Kitada and Koya, 2021). Understanding the underlying mechanisms involved in IR pathogenesis can help us to identify novel therapeutic targets and work towards limiting these health burdens.

As a key regulator of metabolic homeostasis, such as lipid metabolism, energy management, and cell mineral balance, autophagy offers one potential therapeutic target for IR. Mitophagy is a type of selective autophagy that controls mitochondrial mass by removing unnecessary or damaged mitochondria to maintain the stability of the cell environment (Belousov et al., 2021). Abundant evidence indicates that mitophagy plays a critical role in regulating IR and metabolic homeostasis. Defective mitophagy is associated with IR development. Studies have now found that exercise, drugs, and natural products may prevent IR by mediating mitophagy. As such, mitophagy may be a potential new target for treating and improving IR.

### Mitochondrial dysfunction and insulin resistance

The intracellular mitochondrial “energy factories” are involved in the transmission of various complex cellular signals, including those related to energy metabolism, cell proliferation, cell differentiation, cell repair, and cell death (Piantadosi and Suliman, 2017). Although mitochondria can adapt to moderate changes in energy supply and help maintain homeostasis, disturbances in this balance can lead to impaired mitochondrial function. Mitochondrial dysfunction primarily manifests as decreased mitochondrial ATP production, mitochondrial metabolic disorders, such as mitochondrial calcium buffering and ion transport disorders, calcium disorders, mitochondrial DNA (mtDNA) alterations, changes in mitochondrial dynamics, increased production of reactive oxygen species (ROS), and increased cell apoptosis (Lahera et al., 2017). Lack of mitochondria, their functional degradation, and uncoordinated mitochondrial matrix transport all contribute to a bioenergy disorder. Mitochondrial dysfunction is a consequence of many diseases including IR pathogenesis in metabolic disorders such as obesity, metabolic syndrome, and diabetes (Sangwung et al., 2020).

Mitochondrial dysfunction is characterized by increased production of ROS (Sies et al., 2022), an intermediate product of molecular oxygen reduction in the body. Excessive ROS production in the body is called oxidative stress (OS) and refers to a series of reactions mediated by the imbalance between oxidative and antioxidative processes resulting from a disproportionate scavenging capacity (Napolitano et al., 2021). Excessive ROS impair mitochondria by inducing mtDNA mutations, damaging the mitochondrial respiratory chain, altering membrane permeability, and influencing Ca^2+^ homeostasis and mitochondrial defense systems (Li A. et al., 2021). Affected mitochondria are characterized by reduced energy production, reduced fatty acid oxidation, and increased glucose dependence for ATP synthesis (Jakovljevic et al., 2021). Altered mitochondrial function reduces mitochondrial biosynthesis and decreases the rate of fatty acid beta-oxidation in insulin target organs. Changes in the metabolic pathways of adipocytes in obesity, including adipogenesis and fatty acid esterification, result in the accumulation of free fatty acids and fatty intermediate metabolites in adipocytes (Lipina et al., 2013), reduction of adipocyte sensitivity to insulin, and inhibition of insulin signal transduction (Rao et al., 2013), leading to impaired insulin sensitivity (Heinonen et al., 2015). In addition, changes in calcium levels (increased release of Ca2+ from the endoplasmic reticulum and depletion of endoplasmic reticulum Ca2+ stores) (Ly et al., 2017) and decreased mitochondrial membrane potential (Giralt and Villarroya, 2017) lead to impaired oxidative capacity and increased ROS production; this may also result in impaired insulin signal transduction and IR.

### Mitochondrial dynamics and insulin resistance

**FIGURE 1:**
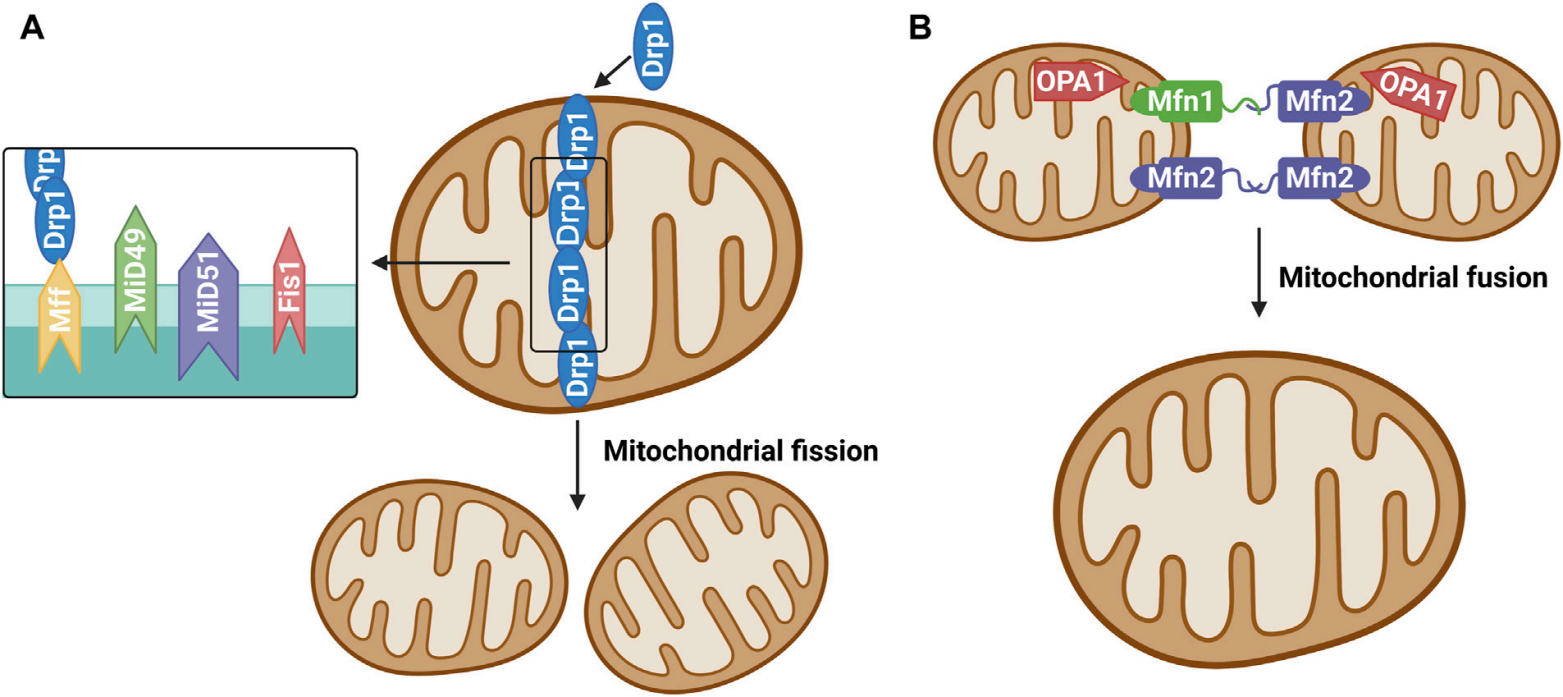
**(A)** Mitochondrial fission. The protein that regulates mitochondrial fission is mainly Drp1, which is generally localized in the cytoplasm under physiological conditions. Various stimulators result in recruitment and oligomerization of Drp1 to mitochondria by binding its receptors Mff, Fis1, MiD49 and MiD51, and then induce mitochondrial fission.**(B)** Mitochondrial fusion. Mitochondrial fusion proteins include mainly mitochondrial fusion protein 1 (Mitofusin1, Mfn1), mitochondrial fusion protein 2 (Mitofusin 2, Mfn2) and optic atrophy one protein (OPA1). Mfn1 and Mfn2 are widely expressed in the mitochondrial outer membrane and mediate the fusion of outer membrane of mitochondria. OPA1 is mainly involved in the mitochondrial inner membrane fusion process.

Mitochondrial dynamics is primarily regulated achieved through post-translational modification of mitochondrial fusion and fission enzymes (Flippo and Strack, 2017). Studies have confirmed that many proteins mediate the mitochondrial fission/fusion process, and mitochondrial dynamin-related protein 1 (Drp1) is among the key proteins involved in mitochondrial fission (Ramonett et al., 2022). During mitochondrial fission, Drp1 is recruited from the cytoplasm to the outer (OMM) under the action of specific cell signals. Drp1 forms a ring structure through oligomization and shrinks the ring structure with the energy provided through its hydrolysis by guanosine-triphosphate (GTP) and divides mitochondria into two progeners by separating the double-membrane structure (Ramachandran, 2018). Studies have divided the mitochondrial fission process into approximately three steps (Lee et al., 2016; McBride and Frost, 2016). First, the endoplasmic reticulum (ER)-actin system triggers the shrinkage of mitochondria. Second, Drp1 located at the ER pre-contraction division site is hydrolyzed by GTP to transform its structure into a spiral and further shrink the mitochondria, but it did not reach the critical point of division. Finally, dynamin 2 (Dyn2), a more efficient mechanical enzyme located in the Drp1 hypersystolic domain, is activated. This enzyme promotes OMM contraction and ultimately leads to mitochondrial fission. Studies on mitochondrial fission models have shown that Drp1 receptors on OMM include fission protein 1 (Fis1), mitochondrial fission factor, and mitochondrial dynamoproteins MiD49 and MiD51. They are all small molecular OMM anchor proteins that can promote the transfer of Drp1 to OMM and promote mitochondrial division (Ramachandran, 2018) (Figure 1A). Mitochondrial fusion requires the coregulation of three proteins: Mfn1, Mfn2, and Opa1. Mfn1 and Mfn2 promote the fusion of OMM, while Opa1 promotes the fusion of the inner mitochondrial membrane (IMM) (Lee et al., 2017; Chan, 2020). Unlike Drp1 and Dyn2, Mfn 1/2 and Opa1 are anchored to the cytoplasm of OMM and IMM, respectively, by transmembrane segments, where Opa1 plays a vital role in maintaining the microtubule morphology of IMM “crest” (Ramachandran, 2018). Mfn2 exists in both the ER and mitochondria and plays a key role in the coordination between the ER and mitochondria (Filadi et al., 2018) (Figure 1B).

Mitochondrial dynamics plays a crucial role in maintaining the morphology, number, distribution, and function of mitochondria. Mitochondria is a highly dynamic organelle. Abnormal mitochondrial fission/fusion promotes increased ROS production, mitochondrial fragmentation, and energy metabolism disorders, which may lead to the development of metabolic diseases, such as IR and decreased insulin sensitivity (Dubé et al., 2020). Studies have shown that mitochondrial fragmentation and fission have been implicated in insulin resistance in skeletal muscle cells (Jheng et al., 2012), whereas overexpression of the fusion factors MFN one and MFN 2 has an insulin-sensitizing effect (Lin et al., 2018). Moore *et al.* (Moore et al., 2019) study showed that endurance exercise is sufficient to induce changes in the mitochondrial life cycle including mitochondrial fission signaling through Drp1, while impaired dynamic flux of mitochondrial remodeling is associated with derangements in metabolism and insulin sensitivity.

### Mitophagy

**FIGURE 2:**
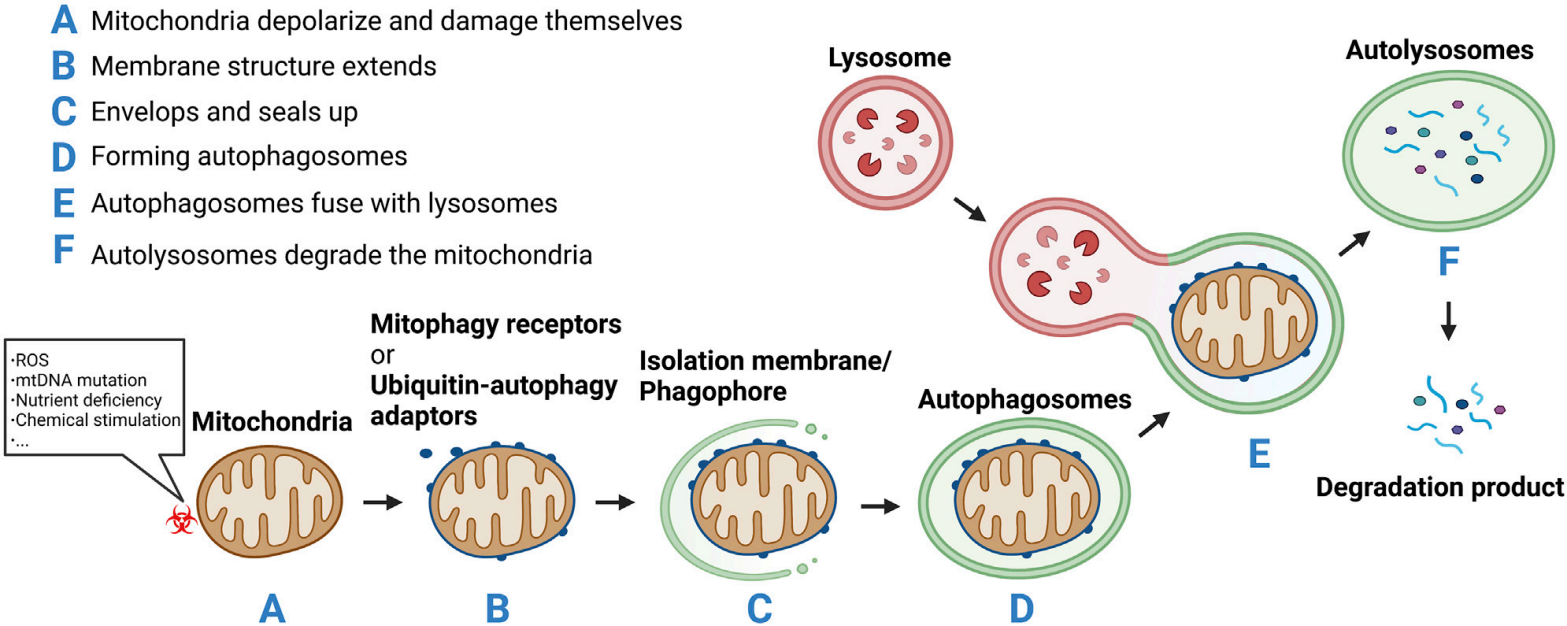
**(A)** Mitochondria depolarize and damage themselves in response to various stimuli such as reactive oxygen species (ROS), nutrient deficiency, chemical stimulation, and mtDNA mutation.**(B)** Mitochondria membrane structure extends.**(C)** Envelops and seals up the mitochondria to be removed.**(D)** Forming autophagosomes.**(E)** The autophagosomes fuse with lysosomes to form autolysosomes. **(F)** The autolysosomes degrade the mitochondria.

Autophagy can be subdivided into microautophagy, macroautophagy, and chaperone-mediated autophagy (CMA) (Klionsky et al., 2021). Microautophagy refers to the direct encapsulation and degradation of cytoplasmic content by lysosomes. Macroautophagy is the most common form of autophagy, wherein a double-layer membrane is formed within the cytoplasm to surround the material to be cleared. This enclosed material is then fused with the lysosome for degradation. In CMA, heat shock protein 70 and its partner molecules fold the substrate protein and transpose it onto the lysosomal membrane for degradation (Liao et al., 2021). Depending on whether the clearance is selective, autophagy is categorized as nonselective or selective autophagy. Nonselective autophagy occurs when the cell is under starvation conditions and degradation products produced by autophagy provide the cell with its most basic nutrients. Selective autophagy occurs when damaged organelles, such as mitochondria, need to be removed. Thus, mitophagy is a form of selective macroautophagy. The concept of mitophagy was first formally proposed by Lemasters in 2005 (Lemasters, 2005). It described a process through which cells selectively remove damaged or unwanted mitochondria in a timely manner. In this process, mitochondria depolarize and damage themselves in response to various stimuli such as ROS, nutrient deficiency, chemical stimulation, and mtDNA mutation. Subsequently, a membrane structure extends, envelops, and seals up the mitochondria to be removed, forming autophagosomes which fuse with lysosomes to form autolysosomes. These autolysosomes degrade the mitochondria (Onishi et al., 2021) (Figure 2). Through mitophagy, damaged or redundant mitochondria are specifically selected and removed, thus maintaining the dynamic equilibrium of the mitochondrial functional network and ensuring its number and function.

Metabolic waste removal is a key aspect of homeostasis. To prevent the accumulation of toxic molecules, to make space for new elements, or to reuse structures, organisms have developed sophisticated systems to degrade and remove substances that are no longer required. Mitophagy is an important part of homeostasis and can be used to remove metabolic waste including specific substrates, such as an unfinished or damaged protein complex, or entire subcellular structures for eventual lysosomal degradation. Mitophagy is the specific degradation of damaged or unwanted mitochondria and is particularly critical for improving IR in metabolic disorders such as obesity, diabetes, and metabolic syndrome.

### Mitophagy signaling pathways involved in insulin resistance

Mitophagy signaling pathways are diverse and complex, and there is currently no consensus about exactly which pathways are involved. Here, we briefly introduce the mitochondrial autophagy initiation pathway AMPK/mTOR and the mitochondrial autophagosome formation pathways ubiquitin-mediated mitochondrial autophagy pathway (PINK1/Parkin) and autophagy receptor-mediated mitochondrial autophagy (FUNDC1 and BNIP3/Nix).

### AMPK/mTOR

**FIGURE 3:**
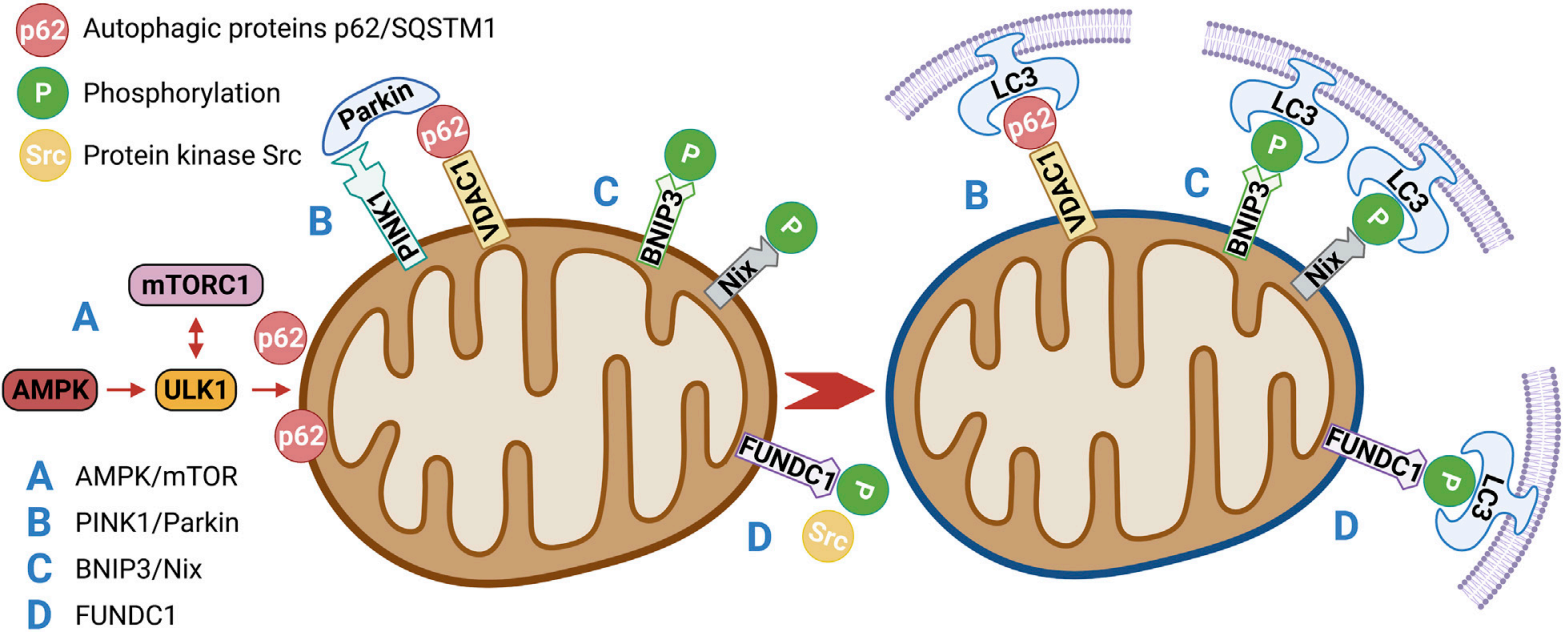
**(A)** AMPK/mTOR is a crucial regulatory pathway of autophagy and often acts as the initiation pathway of mitophagy. Under nutrient-rich conditions, mTORC1 strongly inhibits autophagy through direct ULK1 phosphorylation. When the cell is starved or depleted of energy, mTORC1 dissociates from the complex and ULK1 autophosphorylation increases; AMPK promotes autophagy not only by directly activating ULK1 but also by negatively regulating mTORC1 and blocking its inhibition of ULK1.**(B)** PINK1/Parkin-mediated mitophagy depends on the activity of voltage-dependent anion channels (VDAC1) and P62/SQSTM1, which interact directly with light chain 3 (LC3) to recruit autophagosomes. Damaged mitochondria are enclosed in the autophagosomes and transported to lysosomes for degradation.**(C)** BNIP3/Nix promote mitophagy through interaction with processed LC3-related molecules at nascent phagophores.**(D)** Under hypoxia and other conditions, the activity of protein kinase Src, which inhibits the activity of FUNDC1, will also decrease, so as to enhance the activity of FUNDC1 and promote the interaction with LC3, thus affecting mitophagy.

AMPK is a key physiological energy sensor. It is a major regulator of energy balance in cells and organisms. It coordinates various metabolic pathways, maintains energy homeostasis by regulating glucose and lipid metabolism (Rodríguez et al., 2021), and ultimately regulates cell and organ growth. Dysregulation of the AMPK pathway underlies disease states such as neurological disorders, cardiovascular diseases, cancer, and metabolic disorders (Entezari et al., 2022). The regulation of AMPK energy metabolism is mediated by several related signaling pathways, including the AMPK and mammalian target of rapamycin (mTOR) signaling pathway. The AMPK/mTOR pathway is the switch from anabolism to catabolism in cells. AMPK/mTOR is a crucial regulatory pathway of autophagy and often acts as the initiation pathway of mitophagy. ULK1, a mammalian homolog of yeast protein kinase ATG1, is a conservative substrate of AMPK and is essential for autophagy. In mammals, the absence of AMPK or ULK1 leads to abnormal accumulation of p62, which is a well-known receptor for autophagy, and mitochondrial autophagy defects. mTOR is a central regulator of cell growth and proliferation and forms two complexes: mTORC1 and mTORC2. mTORC1 mainly regulates protein synthesis and the cell cycle and mTORC2 is crucial for actin cytoskeleton and cell survival. mTORC1 is regulated by various signals, such as growth factors, amino acids, and cellular energy, and regulates many important cellular processes, including autophagy. Under nutrient-rich conditions, mTORC1 strongly inhibits autophagy through direct ULK1 phosphorylation (Kim et al., 2016). However, when the cell is starved or depleted of energy, mTORC1 dissociates from the complex and ULK1 autophosphorylation increases; AMPK promotes autophagy not only by directly activating ULK1 but also by negatively regulating mTORC1 and blocking its inhibition of ULK1. Therefore, AMPK-mediated phosphorylation of ULK1 is critical for inducing mitophagy (Trefts and Shaw, 2021) (Figure 3A). Seabright *et al.* (Seabright et al., 2020) recently found that AMPK activation promotes mitophagy by increasing mitochondrial fission and autophagy without the PINK1/Parkin pathway, and AMPK/mTOR activation improves IR, which has been demonstrated in many studies (Wang et al., 2020; Zhu Y. et al., 2021; Su et al., 2021).

### PINK1/parkin

PINK1/Parkin-mediated mitophagy is the most widely and intensively studied pathway in the mammalian system (He L. et al., 2019). PINK1 is a mitochondrial serine/threonine kinase (Ser/Thr), while Parkin is an E3 ubiquitin ligase. Under normal conditions, Parkin is located in the cytoplasm and its E3 activity is inhibited; PINK1 functions upstream of Parkin. PINK1 phosphorylates Parkin and promotes the translocation of Parkin from the cytoplasm to mitochondria for mitophagy (Agarwal and Muqit, 2022). In normal cells, a PINK1 protein synthesized in the cytoplasm is translocated to the inner mitochondrial membrane, cleaved by PARL proteases on the membrane, and rapidly degraded by the ubiquitin–proteasome pathway (Sekine and Youle, 2018). When mitochondria are damaged or mitochondrial membrane potential is lost, PINK1 is no longer cleaved and degraded, but is stably localized on the outer mitochondrial membrane, PINK1 then phosphorylates Parkin directly at a highly conserved residue, Ser65 (Heo et al., 2015; McLelland et al., 2018), that lies within the Ubl (ubiquitin-like) domain of Parkin. Phosphorylation translocates Parkin from the cytoplasm to the outer mitochondrial membrane surface and stimulates Parkin’s E3 ligase activity. PINK1/Parkin-mediated mitophagy depends on the activity of voltage-dependent anion channels (VDAC1) and P62/SQSTM1 (Ivankovic et al., 2016), which interact directly with light chain 3 (LC3) to recruit autophagosomes. Damaged mitochondria are enclosed in the autophagosomes and transported to lysosomes for degradation (Villa et al., 2017) (Figure 3B). However, PINK1 mutation inhibits the translocation of Parkin to mitochondria, which leads to mitophagy defects and possibly results in the accumulation of damaged mitochondria, production of more ROS and less ATP, and induction of apoptosis (Zhang et al., 2019). These results suggest that components of the PINK1/Parkin pathway are molecular contributors to mitophagy. Studies have shown that the expression of the mitochondrial autophagy modulator PINK1/Parkin is significantly increased in patients with prediabetes who may have IR (Frank et al., 2012; Bhansali et al., 2017). In addition to the PINK1/Parkin pathway, other E3 ubiquitin ligases have been found to mediate or function in ubiquitin-mediated mitophagy, such as MULAN (Ambivero et al., 2014), SIAH1 (Szargel et al., 2016), ARIH1 (Villa et al., 2017), and RNF34 (He X. et al., 2019). However, whether these ubiquitin-mediated mitophagy pathways are related to IR remains to be further explored.

### BNIP3/nix

BNIP3/Nix is an important molecular mediator involved in hypoxia or mitophagy induced by mitochondrial membrane depolarization (Li Y. et al., 2021). Nix is an outer mitochondrial membrane protein that interacts with molecules associated with the microtubule-associated protein one LC3 in primitive phagocytes to induce mitophagy to clear damaged mitochondria (Xu et al., 2019). BNIP3, a member of the BCL-2 family, is associated with dynamin-related protein one and optic atrophy one and thus inhibits fusion and promotes mitochondrial fission (Liu K. et al., 2022). BNIP3 and Nix are both hypoxia-inducible genes that encode molecular adaptors that promote mitophagy through interaction with processed LC3-related molecules at nascent phagophores (Chourasia et al., 2015). BNIP3 has been suggested to decrease mTOR activity and independently affect mitophagy by promoting LC3 expression. Therefore, Nix and BNIP3 may be involved in mitophagy (Figure 3C). A novel lipotoxicity-triggered signaling cascade has been found to depend on mitochondrial regulation by BNIP3, leading to desensitization of adrenergic signaling. Adrenergic signaling stimulates cAMP production and subsequently activates PRKA/PKA (protein kinase A, also known as cAMP-dependent protein kinase a), which then inhibits BNIP3 function and restores insulin signaling. Exercise or pharmacological regulation of PRKA/PKA may overcome IR in myocytes (Rosa et al., 2021).

### FUNDC1

In 2013, a novel hypoxia-mediated mitophagy protein was first reported by Liu *et al.* (Liu et al., 2012). FUNDC1 relies on the conservative LC3-interacting region (LIR) domain to bind to LC3 and participate in autophagy (Liu H. et al., 2022). The binding of FUNDC1 to LC3 is inhibited by LIR mutation or deletion. In cells with a deletion of autophagy protein 5 (ATG5), FUNDC1 expression is reduced and mitochondrial autophagy is inhibited, indicating that FUNDC1 is also dependent on ATG5. Under normal conditions, FUNDC1 activity is inhibited by the phosphorylation of protein kinase Src. However, under hypoxic conditions, Src activity decreases and FUNDC1 phosphorylation occurs, leading to enhanced FUNDC1 activity and its interaction with LC3 (Figure 3D). Mice lacking FUNDC1 develop more severe obesity and IR when fed a high-fat diet. FUNDC1 ablation results in defective mitophagy and impaired mitochondrial quality control *in vitro* and in white adipose tissue (Wu et al., 2019). Interestingly, Fu suggested that FUNDC1-mediated mitophagy in skeletal muscle can prevent IR in mice fed a high-fat diet (Fu et al., 2018). These findings suggest that FUNDC1-mediated mitophagy plays a key role in regulating systemic metabolism and may offer a therapeutic target for the management of IR.

### Mitophagy and insulin resistance

Mitophagy is involved in many human diseases, including neurological disorders such as Parkinson’s (Eldeeb et al., 2022) and Alzheimer diseases (Lee S. H. et al., 2022), cardiovascular diseases such as heart failure (Li et al., 2022) and cardiomyopathy (Rabinovich-Nikitin et al., 2021), cancer (Poole and Macleod, 2021), and aging (Guo and Chiang, 2022). Significant interest now exists about the role of mitophagy in IR in metabolic disorders.

Studies have shown that ROS-induced mitochondrial depolarization is an upstream activator of the mitochondrial autophagy pathway PINK1/Parkin (Wang et al., 2012; Wei et al., 2014). Therefore, dysregulation of mitochondrial function can upregulate mitochondrial autophagy, thereby increasing the clearance of damaged mitochondria and enhancing mitochondrial function. As a result, the downstream insulin pathway becomes enhanced and IR symptoms are improved. On the contrary, abnormal mitochondrial autophagy allows for an accumulation of damaged mitochondria and an accumulation of ROS and other metabolic products. This impairs the intracellular insulin signaling pathway and induces IR (Gonzalez et al., 2011). At the same time, AMPK/mTOR phosphorylation can affect energy (ATP) synthesis to achieve apoptosis metabolism, phospholipid metabolism, and glucose metabolism, thus improving IR (Andreozzi et al., 2016; Lv et al., 2018).

These results suggest that oxidative damage-induced mitochondrial dysfunction plays a crucial role in the development of IR. Mitophagy can clear damaged mitochondria and alleviate OS, which in turn alleviates IR. Therefore, improving mitochondrial function through mitophagy is speculated to be a reasonable way to treat IR.

### Mitophagy as a therapeutic target for insulin resistance

Current research is focused on improving mitochondrial function via approaches such as mitochondrial transplantation, antioxidant administration, biofuel administration, mesenchymal stem cell therapy, and induced uncoupling (Koh et al., 2020). However, there is no definite support for the efficacy of these approaches and many unresolved questions remain. Therefore, mitophagy may represent a new potential therapeutic target, especially for improving IR. Although basic science research has made the link between mitophagy and IR, few clinical studies have been conducted.

Exercise may improve IR through mitophagy. Studies have shown that exercise induces mitophagy through Ampk-dependent activation of Ulk1 in skeletal muscle (Laker et al., 2017). Studies have shown that exercise can enhance AMPK activity and activate mitophagy through a series of downstream effects (Gao et al., 2020). In the context of exercise, PTEN-induced mitophagy has been studied as a PINK1/Parkin pathway (Memme et al., 2021). Studies have found that exercise training increases Pink1 mRNA 64 and Parkin protein levels in mouse skeletal muscle (Jamart et al., 2013), and exercise activates the mitochondria-specific autophagy mediator Parkin (Ehrlicher et al., 2020), but these findings are far from conclusive. Laker *et al.* (Laker et al., 2017) found no significant changes in Pink1 protein levels in skeletal muscle after acute exercise. but at present, it is mainly concerned with autophagy in myocardial diseases (Li et al., 2021). The effect of exercise on IR through the PINK1/Parkin pathway is still unclear. In autophagy receptor-mediated mitophagy, endurance exercise can promote the expression of the mitochondrial autophagy protein BNIP3 in skeletal muscle, which means that the exercise adaptation of skeletal muscle cannot be separated from the involvement of mitophagy. Furthermore, experimental data show that both acute exercise and endurance exercise promote BNIP3 gene expression in skeletal muscle (Lira et al., 2013). This suggests that both BNIP3 and Nix are motion-sensitive molecules, and therefore, exercise may be able to remove damaged mitochondria by activating the BNIP3/Nix pathway. Drake *et al.* (Drake et al., 2021) study also found that Ulk1 feedback regulation of AMPK is required for exercise training-induced improvement in skeletal muscle insulin response. Although in a cell culture model experiment, Gao *et al.* (Gao et al., 2020) used electrical stimulation of myotubes as a simulated exercise and found that the mitochondrial outer membrane protein FUNDC1 was induced through the AMPK-ULK1 pathway, thereby initiating mitophagy, direct experiments showing that exercise can initiate the FUNDC1 pathway are lacking. BNIP3-induced impairment of mitophagy and glucose uptake can be reversed through direct BNIP3 phosphorylation by PRKA/PKA, which leads to the translocation of BNIP3 from the mitochondria and sarcoplasmic reticulum to the cytoplasm. These findings provide insights into the role of BNIP3, mitochondrial transition, and insulin signaling in muscle cells damaged by overfeeding when the overall autophagy-related gene expression is reduced. PRKA/PKA can overcome the mechanism of IR in muscle cells through exercise activation (Rosa et al., 2021). Exercise-induced autophagy was also initiated through the FUNDC1 pathway (Gao et al., 2020). The HE (He et al., 2012) study showed that exercise can upregulate the autophagic activity of skeletal muscle, liver, and other tissues in wild mice and improve hyperglycemia, hyperlipidemia, and leptin resistance induced by a long-term high-fat diet. Thus, activation of autophagy is critical for exercise-induced improvements in energy metabolism; exercise can enhance insulin sensitivity by activating autophagy. Therefore, exercise may induce PINK1/Parkin, BNIP3/Nix, and FUNCC1 pathways to participate in exercise adaptation via different mitophagy pathways, which may be beneficial for improving IR.

Some existing drugs, including metformin, liraglutide, and sitagliptin, may affect mitophagy. One of the oldest first line antidiabetic drugs, metformin, is an AMPK activator that modulates mTOR (Zhu et al., 2021). In a study of monocytes, metformin activated AMPK-induced mitophagy to restore normal mitochondrial function, thereby improving pancreatic beta cell function and IR (Bhansali et al., 2020). Glucagon-like peptide-1 (GLP-1) receptor agonists can activate the GLP-1 receptor, enhance insulin secretion in a glucose-dependent manner, inhibit glucagon secretion, delay gastric emptying, and reduce food intake through central inhibition of appetite, thereby achieving hypoglycemic effects (Laurindo et al., 2022). Liraglutide, an important GLP-1 receptor agonist, may play a key role in maintaining mitochondrial homeostasis by regulating PINK1/Parkin-mediated mitophagy and inhibiting PINK1/Parkin overexpression (Zhang et al., 2022). However, PINK1/Parkin-mediated mitophagy pathway is associated with IR. Dipeptidyl peptidase-4 inhibitor (DPP-4) can inhibit the inactivation of GLP-1 and the glucose-dependent insulin-stimulating polypeptide (GIP) (Florentin et al., 2022), and thus act as a hypoglycemic agent. Sitagliptin is one of the first DPP-4 inhibitors on the market, and sitagliptin can effectively improve the progression of obesity-induced IR and liver steatosis by enhancing autophagy through the AMPK/MTOR pathway (Zheng et al., 2018). These studies on novel mechanisms through which existing drugs affect mitophagy via various pathways may broaden ideas for IR treatment.

Some products that are found in nature can also induce mitophagy and thus improve IR. Zhu *et al.* (Zhu Z. et al., 2021) newly published study provides a new axis for the antidiabetic activity of astragaloside IV (AS-IV), a compound found in the medicinal herb *Astragalus membranaceus*. AS-IV administration is associated with AMPK phosphorylation and subsequent reduction in mTOR expression, which then induces autophagy and thus improves IR and prevents liver injury in type 2 diabetes. Quercetin, a flavonol compound, is widely distributed in the plant kingdom and has various biological activities. Quercetin attenuates ROS production, ATP synthesis, and mitochondrial damage in a high-glucose-exposed rat Schwann cell line and streptozocin-induced diabetic rats by stimulating the AMPK/PGC-1α pathway (Zhang et al., 2021). In addition, quercetin has been reported to improve triglyceride accumulation through fatty acid synthase regulation, which restores mitochondrial mass by activating AMPK to regulate mitophagy. Quercetin is associated with inhibition of inflammation and IR in mouse adipose tissue (Herranz-López et al., 2020). Berberine, a quaternary ammonium compound isolated from Rhizoma coptidis and Cortex Phellodendri. Berberine was found to restore mitochondrial ROS production, mitochondrial dysfunction, and fatty acid oxidation failure in the diabetic nephropathy mouse model, and thus regulate glucose and lipid metabolism, reduce oxidative damage, and improve insulin sensitivity (Qin et al., 2020). Berberine can reduce mitochondrial numbers via AMPK signal pathway-dependent mitophagy activation (Hang et al., 2018). Other studies have found that palmitic acid can reduce mitochondrial fusion and increase mitosis. Puerarin, an isoflavone found in several plants, counteracts these changes; this can improve the palmitic acid-induced disequilibrium kinetics of fusion and division. In addition, palmitic acid impairs mitophagy by decreasing autophagic flux, resulting in decreased mitochondrial PINK1 and Parkin levels and eventual apoptosis (Chen et al., 2018). By contrast, puerarin promotes PINK1/Parkin-mediated mitophagy to remove dysfunctional mitochondria (Vives-Bauza et al., 2010). Therefore, puerarin can attenuate palmitic acid-induced cell apoptosis. However, the detailed mechanism underlying puerarin-induced cell-protective mitophagy needs further exploration. Puerarin inhibits palmitic acid-mediated endothelial cell inflammation, thereby reducing endothelial IR (Huang et al., 2012). Interestingly, oral carbohydrate administration preoperatively has been found to reduce IR in patients after colorectal surgery by stimulating the AMPK/mTOR pathway (Shi et al., 2020); however, whether mitophagy is involved in the regulatory mechanism remains to be explored.

The aforementioned methods may be beneficial to IR pathogenesis and progression (Table 1). However, long-term use of certain natural products and carbohydrate for improving IR may cause potential side effects. At the same time, it is important to recognize that mitophagy can be a double-edged sword. Normal mitochondrial autophagy forms a defense mechanism against damaged mitochondria and has been shown to protect cells such as neurons and cardiomyocytes. However, excessive mitophagy can lead to abnormal mitochondrial circulation, energy metabolism disorders, harmful effects on cell homeostasis, and even cell death. Thus, a carefully titrated reduction of mitosis and restoration of normal mitophagy is going to be the ultimate goal for improving IR.

**TABLE 1:** Models and signaling pathways of factors influencing in mitophagy.

Groups	Methods	Experiment model	Signaling pathway	References
Physical	Exercise	Ulk1- iMKO and the wild type littermate mice	AMPK	#808080; Drake et al., 2021
Drug	Metformin	Peripheral blood mononuclear cells (PBMCs)	AMPK/mTOR	#808080; Bhansali et al., 2020
	Liraglutide	The human umbilical vein endothelial cells (HUVECs)	PINK1/Parkin	#808080; Zhang et al., 2022
	Sitagliptin	Male leptin-deficient homozygous ob/ob T2DM obese mice	AMPK/mTOR	#808080; Zheng et al., 2018
Natural products	Astragaloside IV	Male Sprague-Dawley ratsa	AMPK/mTOR	#808080; Zhu et al., 2021a
	Quercetin	The 3T3-L1 cell line and hypertrophic adipocyte	AMPK	#808080; Herranz-López et al., 2020
	Berberine	H9C2 cell line	AMPK	#808080; Hang et al., 2018
	Puerarin	Rat L6 skeletal muscle cells	PINK1/Parkin	#808080; Chen et al., 2018
Other	Carbohydrate	Patients undergoing elective open colorectal cancer resection	AMPK/mTOR	#808080; Shi et al., 2020

AMPK, Adenosine 5′-monophosphate (AMP)-activated protein kinase; mTOR, Mamalian target of rapamycin; Parkin, ubiquitin ligase; PINK1, PTEN-induced putative kinase 1; Bnip3, Bcl-2, 19-kDa interacting protein three; NIX, NIP3-like protein X; FUNDC1, FUN14 domain containing 1.

## Summary and future perspectives

Being an essential semi-autonomous cell organelle, the primary function of mitochondria is to be the cell’s power source. Mitochondria maintain most of the cell’s energy requirements through oxidative phosphorylation and ATP synthesis (Pizzo, 2021). Mitochondrial dysfunction underlies many metabolic abnormalities, such as IR. The vital role of mitophagy in the maintenance of cell homeostasis has recently received increasing attention. This is because normal mitophagy has an active role in the clearance of dysfunctional or structurally damaged mitochondria, especially in the field of pathophysiology.

Recent advances have improved our understanding of the mechanisms regulating AMPK/mTOR-, PINK1/Parkin-, BNIP3/Nix-, and FUNCC1-mediated mitophagy. Mitophagy-mediated through these pathways can play an active role in the clearance of damaged mitochondria in IR. Studies have also shown that some induction interventions can improve IR through mitophagy, including exercise, clinically used hypoglycemic agents such as metformin, liraglutide, and sitagliptin, and some natural products such as astragaloside IV, quercetin, berberine, and puerarin. The aforementioned pathways and induction methods offer novel targets for IR therapy and drug development; however, some underlying mechanisms have not been fully elucidated. However, excessive induction of mitophagy also poses risks for cell homeostasis; therefore, careful regulation of mitophagy to improve IR is desirable.

In conclusion, mitochondrial dysfunction is associated with IR, and some induction interventions have been reported to improve IR by influencing mitophagy through multiple pathways. This may provide new targets and new ideas for IR treatment and the development of drugs.
